# Naturally Occurring Microbiota in Dengue Vector Mosquito Breeding Habitats and Their Use as Diet Organisms by Developing Larvae in the Kandy District, Sri Lanka

**DOI:** 10.1155/2020/5830604

**Published:** 2020-10-12

**Authors:** H. A. K. Ranasinghe, L. D. Amarasinghe

**Affiliations:** Department of Zoology and Environmental Management, Faculty of Science, University of Kelaniya, Dalugama, Kelaniya, Sri Lanka GQ 11600

## Abstract

Naturally occurring microbiota in mosquito larval habitats are among biotic factors which affect the population dynamics of developing larvae. Many microbiota species serve as food items for vector mosquito larvae, and food limitations within habitats adversely affect larval survival, developmental rate, adult fitness, and thereby vector competence. Therefore, identification of microbiota as associates with larvae reveals their relationship between each other as parasites, pathogens, epibionts, or diet organisms. Analysis of associated microbiota species in the dengue vector larval breeding habitats (*n* = 40) and the mosquito larval gut content were conducted in Kandy District in Sri Lanka. Study revealed that a total of 22 microbiota species belong to nine phyla (Amoebozoa, Bacillariophyta, Ciliophora, Chlorophyta, Sarcodina, Cyanobacteria/Cyanophyta, Euglenozoa, Ochrophyta/Heterokontophyta, and Rotifera) were encountered from different *Ae. aegypti* mosquito breeding habitats while 26 microbiota species that belonged to ten phyla were recorded from *Ae. albopictus* mosquito breeding habitats with one additional phylum Arthropoda. Considering *Ae. aegypti* breeding habitats, only *Philodina citrina* in low roof gutters existed as constant species. Considering *Aedes albopictus* breeding habitats, *Volvox aureus* in plastic containers, *Lecane luna* in coconut shells, *Phacus pleuronectes* in concrete slabs, and *Pinnularia* sp. in tree holes existed as constant species. The rest of the microbiota existed as common or accidental/rare species in a variety of habitat types. The Shannon-Weiner diversity (21.01 and 19.36) and gamma diversity (eight and eight) of the microbiota associated with *Ae. aegypti* and *Ae. albopictus* larvae, respectively, in ponds were found to be higher than other types of breeding habitats recorded during the study. Twelve microbiota species were recorded from larval gut analysis as food organisms of both species of mosquito larvae. However, the distribution of gut microbiota species differed between *Ae. aegypti* and *Ae. albopictus* (Chi − square = 21.294, *P* = 0.002). Identification of microbiota as food items of vector mosquito larvae led to a focus on larval food limitation by introducing food competitors, which could be a potential additional tool for integrated vector control approaches within the country.

## 1. Introduction

In terms of public health, mosquitoes are the most important vectors for diseases, and therefore, studying their ecological and environmental conditions influencing their abundance is important. Mosquito habitat ecology plays an important role to determine the larval densities and species assemblage in a particular breeding habitat [1, 2]. Different types of aquatic habitats are utilized by mosquitoes for oviposition, and many mosquito species tend to select both natural and artificial containers as breeding places [3, 4]. In Sri Lanka, dengue has become a significant socioeconomic and public health burden and *Aedes aegypti* and *Aedes albopictus* are widely adapted to urban and suburban environments, acting as vectors of dengue within the country [5]. Water-holding containers were found to be the main larval habitats for *Ae. aegypti* and *Ae. albopictus.* Chan et al. [6] have stated that *Ae. aegypti* breed in indoor-type breeding habitats such as earthenware jars, tin cans, ant traps, rubber tires, bowls, and drums. Immature forms of *Ae. albopictus* prefer artificial containers with stagnant water and additionally found in tree holes, rock holes, hollow bamboo stumps, and leaf axils [7].

Mosquito distribution, abundance, and individual fitness in breeding habitats are known to be dependent on mainly two factors: biotic and abiotic factors. Biotic factors included the interaction of larvae with other associated macrobiota or microbiota taxa [8–10]. When considering the biotic factors associated with mosquito taxa, there is a diversified naturally occurring microbiota assemblage in mosquito breeding habitats which act as partly potential food organisms, controphic species, competitors, parasites, and/or potential mosquito predators, especially microcrustaceans. Species that belonged to Cladocera, Calanoida, Harpacticoida, Cyclopoida, and algae, bacteria, and protists are also found as microbiota associated with different species of mosquito larvae ([11–15]

Nutritional requirements of immature mosquitoes are acquired through the consumption of both dead and living organic material [16]. The larval food items include heterotrophic microorganisms such as bacteria, fungi, and protists which make the largest portion of diet in larvae and organic matter from the environment such as plant debris. Therefore, there are many naturally occurring microbiota associated with larvae of which some may act as food items for them. The availability of suitable and sufficient food sources determine the proliferation of mosquitoes. Further, the quality and quantity of larval nutrition directly influence immature survivorship and their developmental rate which can ultimately alter the adult traits and population dynamics of larvae, such as larval survival rate and growth rate. Therefore, the mosquito population specially developed in container habitats can be regulated by the availability of food resources [17].

Very little information focusing the microbiota species association with vector mosquito breeding habitats are documented from studies conducted in Sri Lanka (Bambaradeniya et al., 2004; [18–20]). Therefore, the present study was conducted to identify naturally occurring microbiota species associated in a variety of dengue vector mosquito breeding habitats in Kandy District in Sri Lanka.

## 2. Methodology

### 2.1. Study Area

Kandy District consists of the extent of land about 1940 km^2^ in the Central Province of Sri Lanka. It is located in high elevated mountainous and thickly forested interior of the island. This has led to relatively wetter and cooler temperature with an average annual precipitation of 2083 mm and annual temperature of 24.5°C. At present, Kandy District is the fourth-highest risk area for dengue transmission in the country, contributing to 8.51% (*n* = 8940) of the total dengue cases reported from the whole country in 2019 [21].

### 2.2. Sampling of Mosquito Breeding Habitats for Microbiota and Mosquito Larvae

**Figure 1:**
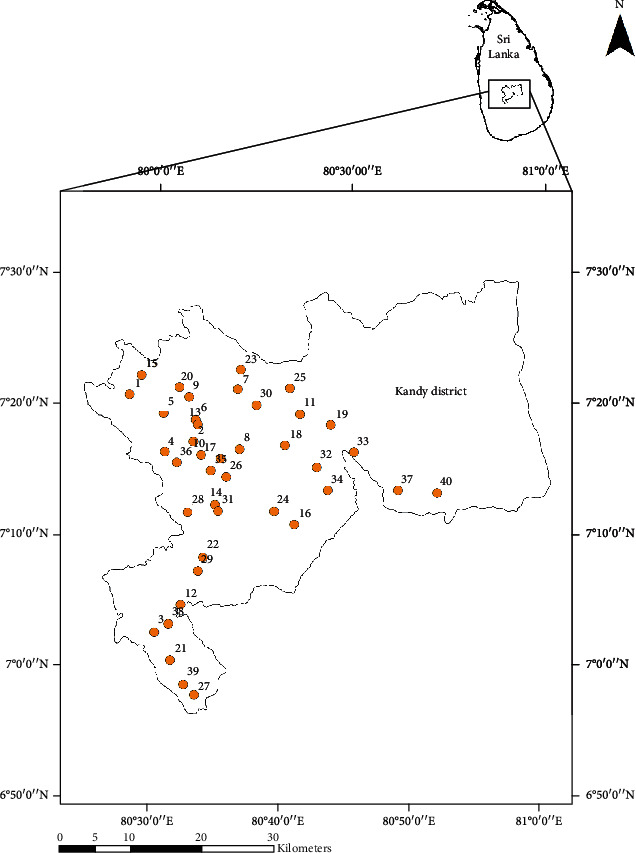
Sampling locations from the selected study site: Kandy District.

A total of forty mosquito breeding habitats were randomly sampled bimonthly between January and August 2019, and each sampling site was georeferenced (GARMIN-etrex SUMMIT) (Figure 1). A standard 250 mL dipper was used to collect water. When dipping was not possible, sampling was done using pipetting or siphoning methods (maximum 250 mL) into a larval rearing container (height 12 cm, diameter 6.5 cm). Five to eight mature larvae were carefully separated into a glass vial with 70% ethanol and labeled for mosquito species identification, and larvae in each sample were identified into species level using standard identification keys [22–25]. Samples positive with *Aedes* larvae were selected.

Water sample collected from each breeding habitat was equally decanted into three plastic containers (6.5 cm width, 12 cm height) during sampling. Two of them were immediately preserved, one using Rose Bengal (5% formalin with 0.04% Rose Bengal stain) solution, and the other using 5% Lugols' solution, for microbiota identification. The remaining sample with mosquito larvae was kept as nonpreserved and covered with a small-sized mesh net for mosquito larval gut analysis study. All samples were labeled and transferred carefully into the laboratory for further processing.

### 2.3. Identification of Microbiota

One milliliter aliquot of preserved sample was examined under the compound microscope (×100 magnification) (OLYMPUS x C21) using a Sedgwick rafter (S-R) cell (50 mm length, 20 mm width, and 1 mm depth) and HYDRO-BIOS phytoplankton chamber (dimensions: 33 × 33 mm, thickness: 1 mL) for quantifying the microbiota. Microbiota species/taxa were identified to a species/taxa level using temporary slide mounts. Mean numbers were calculated from ten replicate observations. Microbiota were identified using standard identification keys (×400 magnification) [26–28].

### 2.4. Determination of Food Habits of Mosquito Larvae Using Gut Analysis

*Aedes aegypti* and *Ae. albopictus* larvae collected from different habitats (plastic/metal containers, shallow ponds, coconut shells, gutters, tires, tree holes, polythene waste, concrete slabs, glassware, and abandoned wells) were individually transferred into small beakers separately, then larvae of third instar and fourth instar stages were selected (*N* = 50), and they were placed in a separate watch glass with 70% ethanol. After 10 minutes, larvae were transferred into another watch glass with distilled water and were kept for 5 minutes in there. Then, they were transferred into another watch glass, and the gut content of each larvae was teased from the membrane using entomological pins into a drop of water and glycerol kept on a slide, while being observed under low-power microscope. Gut contents of those 50 larvae were pooled per one trial, and they were dissolved in 2 mL of 5% formalin.

A microscopic slide was prepared from the gut solution and was observed under the high-power (×400) microscope to identify different food items. One milliliter of gut solution was taken from a dropper and placed in Sedgewick rafter cell and observed under ×100 magnification. Counting was repeated three times, and the mean number of each food item was recorded.

### 2.5. Data Analysis

Occurrence frequencies of microbiota species were categorized as constant for species found in more than 50% of the collections, common when found between 25% and 50% of the collections, and besides, accidental or rare species for less than 25% of the collections [29]. Microbiota alpha (*α*) medium was calculated as the average between the *α* diversity for the system/habitat of the same type; gamma diversity (*ϒ*) was estimated using the total number of species from all samples.

Beta diversity (*β*) was estimated by measuring the species turnover using the *β* − 1 index [30]. It measures the amount in which the regional diversity exceeds the mean alpha diversity and is calculated by the formula *β* − 1 = [(*S*/*α*mean) − 1]/[*N* − 1] × 100.where *S* is the regional diversity or total richness (the number of species per each sampling site), *α*mean is the mean alpha diversity (mean number of species) for each site in each period, and *N* is the number of sites of the period.

Beta diversity over 50% indicates high heterogeneity in microbiota composition among systems, between 20 and 50% indicates intermediate heterogeneity, and below 20% indicates low heterogeneity [30, 31].

The microbiota species diversity was also estimated according to the indices of Shannon and Wiener [32] and evenness [33] as indicated below.

(1) $$\begin{array}{c} \text{Shannon Index}\left(H\right)=-\sum p_{i}\;\ln\;p_{i}, \end{array}$$

where *p*_*i*_ is the proportion (*n*/*N*) (*n*: individuals of one particular species found, *N*: total number of individuals found).

(2) $$\begin{array}{c} \text{Pielo}{\text{u}}^{\text{'}}\text{s evenness}\left(J\right)=H^{\prime }/H_{\max}. \end{array}$$

Pielou's evenness compares the Shannon-Wiener diversity value (*H*′) to the maximum possible diversity value (*H*_max_).

The Chi-square test of independence was used to evaluate the significance of the abundance of different microbiota species as food items of *Ae. aegypti* and *Ae. albopictus*.

## 3. Results

### 3.1. Diversity and Occurrence of Microbiota Species

**Figure 2:**
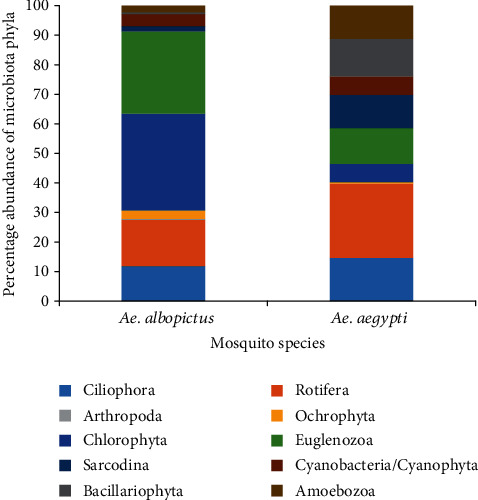
Percentage abundance of microbiota phyla encountered from different mosquito breeding habitats of *Ae. aegypti* and *Ae. albopictus*.

A total of 22 microbiota species belonging to nine phyla (Amoebozoa, Bacillariophyta, Ciliophora, Chlorophyta, Sarcodina, Cyanobacteria/Cyanophyta, Euglenozoa, Ochrophyta/Heterokontophyta, and Rotifera) were encountered from twenty different mosquito breeding habitats of *Ae. aegypti*, while 26 microbiota species that belonged to ten phyla were recorded from twenty breeding habitats of *Ae. albopictus* with one additional phylum Arthropoda (Figure 2). The highest percentage abundance of microbiota was found from phylum Rotifera (25.07% of total microbiota) and phylum Chlorophyta (32.73% of total microbiota) in *Ae. aegypti* and *Ae. albopictus* breeding habitats, respectively.

**Figure 3:**
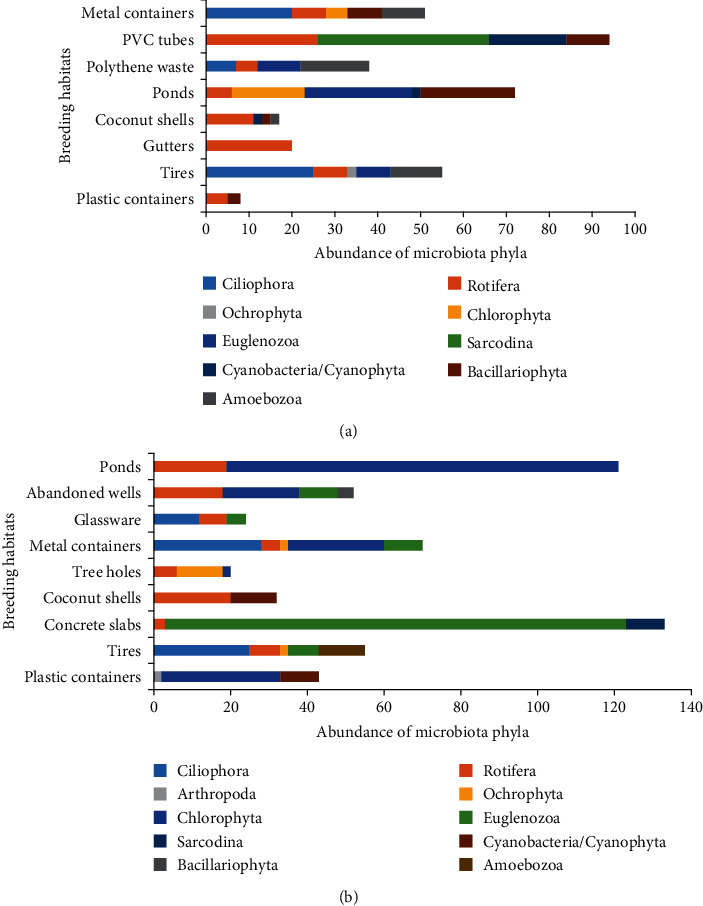
(a) Occurrence of microbiota phyla encountered from *Ae. aegypti* mosquito breeding habitats. (b) Occurrence of microbiota phyla encountered from *Ae. albopictus* mosquito breeding habitats.

Both *Ae. aegypti* and *Ae. albopictus* were commonly captured from temporary micro-breeding habitats, such as plastic and metal containers, low roof gutters, coconut shells, glassware, tires, ponds, and polythene waste. Besides, *Ae. albopictus* was found from tree holes and abandoned wells in the district. No coexisting of *Ae. aegypti* and *Ae. albopictus* was found from the samples during the present study. Majority of habitats occupied by *Ae. aegypti* exhibited a diversity of microbiota belonging to four or five phyla, with common occurrence of phylum Rotifera (Figure 3(a)). Rotifers exhibited a wide range of morphological variations. From the total rotifer population, *Philodina citrina* and *Monostyla bulla* had the abundance of 51.8% and 17.7%, respectively. It is interesting to note that roof gutters occupied by *Aedes* larvae served as shelters only for rotifer population, comprised solely of *Philodina citrina*. Ponds were identified as the breeding habitat with the highest number of microbiota species occurrence (Table 1). Considering the breeding habitats occupied by *Ae. albopictus*, chlorophytes showed the highest abundance, and rotifers were recorded as the common biota, except for plastic containers (Figure 3(b)). However, water-retained concrete slabs occupied by *Ae. albopictus* larvae were highly dominated by *Phacus pleuronectes* (phylum Euglenozoa) (90.2%) (Table 1). Microbiota species of the phylum Arthropoda had the lowest abundance, and its species richness was recorded as one species.

**Figure 4:**
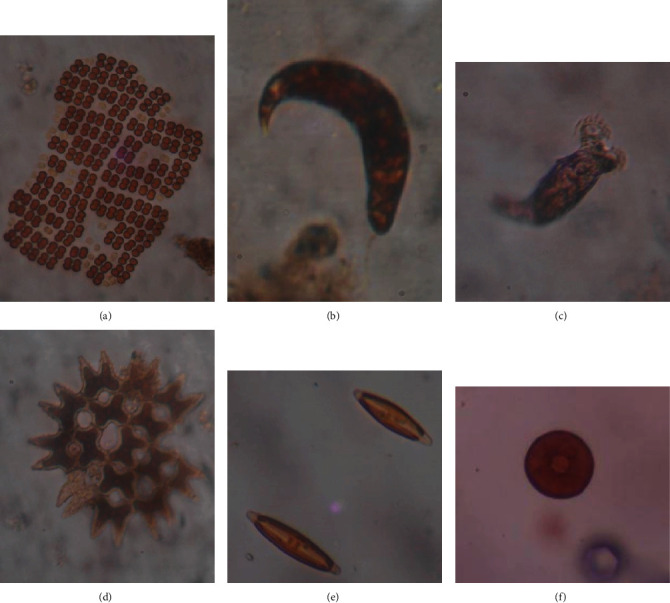
Microscopic view of some microbiota species encountered from mosquito breeding habitats, ×400 magnification. (a) *Crucigenia rectangularis*. (b) *Euglena acus*. (c) *Philodina citrina*. (d) *Pediastrum biradiatum*. (e) *Pinnularia* sp. (f) *Arcella arenaria*.

The microbiota species recorded from different phyla exhibit a wide range of morphological variations (Figure 4). In view of *Ae. aegypti* breeding habitats, only *Philodina citrina* (100%) in gutters existed as constant species in the particular breeding habitat. All the other microbiota existed as common or accidental/rare species in the habitat type according to their abundance (Table 1). In contrast, *Volvox aureus* (72.1%) in plastic containers, *Lecane luna* (62.5%) in coconut shells, *Phacus pleuronectes* (90.2%) in concrete slabs, and *Pinnularia* sp. (60%) in tree holes were found as common species in *Ae. albopictus* breeding habitats. However, the majority of the microbiota were found as common or accidental/rare species in the habitat type according to their abundance in each breeding habitat (Table 1).

The highest species richness and Shannon-Weiner diversities of the microbiota associated with both *Ae. aegypti* larvae for a habitat were found from ponds (Table 2, gamma diversity). The same observation was taken for *Ae. albopictus*. These ponds were small-sized with floating garbage. Tires for *Ae. aegypti* larvae and metal containers for *Ae. albopictus* larvae had the highest beta diversity, indicating higher heterogeneity in microbiota composition. The plastic containers, coconut shells, and polythene waste habitats had a beta diversity between 20 and 50%, indicating intermediate heterogeneity in microbiota composition among the systems. This observation corresponded to ponds, metal containers, and concrete slab breeding habitats from larvae of *Ae. albopictus*. The rest of habitats were with a beta diversity below 20%, indicating low heterogeneity.

### 3.2. Identified Microbiota as Food Items to Mosquito Larvae

**Figure 5:**
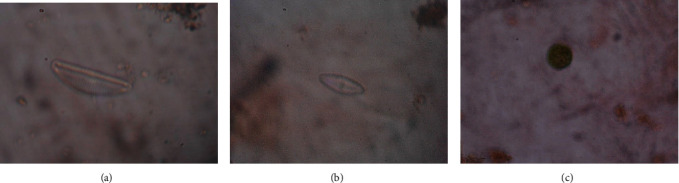
Microscopic view of some food items encountered from mosquito larval gut, ×400 magnification. (a) *Cymbella affinis*. (b) *Pinnularia* sp. (c) *Chlorella* sp.

Only twelve microbiota species were identified from the gut of *Aedes aegypti* and *Ae. albopictus* (Table 3). The identified microbiota (Figure 5) belonged to six phyla. The majority of the food particles inside guts were detritus. Moreover, nondigested insect and/or crustacean parts were also observed. The preferred microbiota species as food items of larvae included species belonging to different genus such as *Cymbella*, *Gomphonema*, *Synedra*, *Closterium*, *Cosmarium*, *Chlorella*, *Volvox*, *Oscillatoria*, *Anabaena*, *Spirulina*, *Phacus*, and *Pinnularia*.

From the microbiota species encountered in *Aedes aegypti* larval gut, Bacillariophyta (38.98%), Cyanobacteria/Cyanophyta (22.03%), and Ochrophyta (20.34%) comprised the majority, while the rest consisted Charophyta (11.86%) and Euglenozoa (6.79%). However, the gut content of *Ae. albopictus* larvae mainly belonged to Chlorophyta (40.62%), while the rest to Cyanobacteria/Cyanophyta (15.63%), Bacillariophyta (3.13%), Euglenozoa (3.12%), Ochrophyta (15.62%), and Charophyta (21.88%). Statistics of the Chi-square test of independence (Chi − square = 21.294, *P* = 0.002) indicated that the distribution of gut microbiota species in both *Ae. aegypti* and *Ae albopictus* differed significantly at a 95% level of confidence.

## 4. Discussion

The relationship between the associated naturally occurring microbiota in the larval habitats and the food habits of mosquito larvae was detected through a gut analysis. The present study results revealed that there are microbiota species associated with larvae which serve as preferred food items for them. But, the larvae are not selecting all the microbiota species as food items in which the microenvironment offers. Only a few number of microbiota species serve as food items for larvae, among microbiota species recorded from the breeding habitats. This may be due to the selectivity of food items by larvae and unidentifiable nature of some food items due to full/partial digestion of them within the larval gut. The gut analysis of *Ae. aegypti* and *Ae. albopictus* revealed that they may not tend to select species belonging to genus such as *Monostyla*, *Lecane*, *Philodina, Notholca*, *Diurella*, *Euchlanis*, *Brachionus*, *Colpoda*, *Cephalodella*, *Arcella*, *Acanthocystis*, *Pediastrum*, *Siurella*, *Crucigenia*, and *Alona* as food items.

The present data revealed that the majority of larval food items of *Ae. aegypti* belong to phylum Bacillariophyta (38.98%), while it was from phylum Chlorophyta (40.63%) for *Ae. albopictus*. Marten [34] found that algae are generally represented in the gut of mosquito larvae in proportion to their abundance among the microflora and microfauna in larval habitats as was proved by the present study results. By knowing the kind and composition of food that makes habitat particularly favorable for *Aedes* mosquito survival, it might be possible to manipulate those habitat types to eliminate mosquito breeding. Introduction of food competitive organisms, indigestible or toxic microbiota for larvae as breeding habitats, is a possible way to reduce the survival of larvae. Further, this could be applicable as an additional tool for integrated vector control approaches. In line with this, the present data also showed that food items represented in the larval gut are related to the proportion of their abundance in the larval habitats. Distribution of gut microbiota species in both *Ae. aegypti* and *Ae albopictus* differed significantly in the present study findings, suggesting that two *Aedes* species studied are having different food habitats. This was stated earlier studying the gut contents of *Aedes triseriatus* and *Anopheles quadrimaculatus* and was found different quantitatively and qualitatively [35]. During the gut analysis of *Aedes* larvae, we found detritus as a major food source for both larvae. In artificial containers, the decaying leaf is the main source of organic matter where the habitats associated with vegetation organic matter content are higher [36].

Both *Aedes aegypti* and *Ae. albopictus* larvae were strongly associated with the naturally occurring algae in their habitats in Kandy District. Many algal species were identified as food for larvae in higher proportions for both *Ae. aegypti* and *Ae. albopictus* larvae. However, some studies stated that while most of the algal species are nutritious food for many species of mosquito larvae, few species are able to kill the larvae if ingested in large quantities. Especially Cyanobacteria, the blue-green algae, are able to affect larval mortality by being toxic. Besides, some species of green algae in the order of Chlorococcales are able to kill larvae by being indigestible [37, 38]. However, such toxic cyanobacteria were not encountered during the present study.

Laired (1988) reported that larval food consists of a range of both nonliving material and living organisms including rotifers and crustaceans. Even though many rotifer species were recorded from both *Ae. aegypti* (25.07% of total identified microbiota) and *Ae. albopictus* (15.64% of total identified microbiota) breeding habitats, none was recorded as diet organisms in this study. However, ciliates, protists, and rotifers, singly or in combination, were found to inhibit larval growth instead of serving as food resources. They are able to alter other microbial populations in mosquito breeding habitats and thereby inhibit the *larval* growth. Besides, they can compete with early instar mosquito larvae for obtaining food items [39].

Anyhow, cyanobacteria were recognized in considerable proportions from both *Ae. aegypti* and *Ae. albopictus* larvae during the present investigation, and the number of cyanobacterial cells ingested and digested by larvae was found to be dependent on the cyanobacterial strain and varied with the mosquito species associated [40]. *Anabaena affinis* was the cyanobacterial strain found from the gut of both *Ae. aegypti* and *Ae. albopictus*, and higher cyanobacteria abundance was observed from temporary container breeding habitats. Marten [34] has reported that many species of *Scenedesmus* were found to kill the larvae. However, a recent study by Garcia-Sánchez et al. [41] stated that *Scenedesmus* species were encountered from both larval gut and larval habitats, but its larvicidal property is yet to be confirmed. During the present study, *S. armetus* and *S. bijuga* were found associated with *Ae. albopictus* larval habitats, and *S. quadricauda* were associated with *Ae. aegypti* larval habitats. However, no *Scenedesmus* species were found from larval gut of both species as a food item.

Ecological characterization of *Aedes aegypti* larval habitats in artificial water containers in Girardot, Colombia, revealed the presence of three main taxa of algae: Bacillariophyceae, Chlorophyceae, and Cyanobacteria. Moreover, *Oscillatoria*, *Dactylococcopsis*, *Nostoc*, *Synedra*, *Scenedesmus*, *Pinnularia*, *Cymbella*, *Meridium*, *Navicula*, and *Dictyosphaerium* were also identified in that country [41]. *Synedra*, *Scenedesmus*, and *Pinnularia* were also identified from the *Aedes* breeding habitats in the present study. Furthermore, only some rotifers were found by these authors as zooplankters in very small numbers and in only a few samples [41]. However, the present study findings revealed the presence of many number of rotifers from breeding habitats of both *Aedes aegypti* and *Aedes albopictus* with considerable proportions, especially from *Aedes aegypti* breeding habitats (25.07%). The geographical distribution of microbiota species also varied depending on climatic and environmental conditions. During the present study, *Cymbella affinis*, *Cosmarium quadrum*, and *Oscillatoria* sp. were found in gut contents but not in the water medium. Those microbiota species may retain in the breeding habitat with a lower abundance, thus may not be captured in the collected representative water samples. The larvae inhabit the particular habitat which have fed on those microbiota may captured for the gut analysis, and therefore, those microbiota have identified during the gut analysis of larvae.

The abundance of *Aedes* spp. was affected by the presence of microcrustaceans, such as *Ceriodaphnia* spp., *Chydorus* spp., *Daphnia* spp., *Simocephalus* spp., Calanoida, and larvae of Chironomidae, as they compete efficiently with mosquito larvae for food resources [42]. Besides, larval food competing microbiota introduction to habitats led to larval food limitation and could be a potential additional tool for integrated vector control approaches within the country. Therefore, knowledge on microbiota species naturally associated with larval habitats of a specific geographical location and selection of them as food items for larvae is important. This would lead to determine the potential effects or any adverse effect on other associated microbiota by introducing such food competing organisms into that particular ecosystem. Meanwhile, this type of entomological study also facilitates awareness about the vector breeding places in the local community, which is one of the main factors that determine the success of a vector control program through source reduction. Allocation of the annual budget in the health sector for preventive measures of dengue within the country is considerable because dengue is one of the major public health problems within the country. Furthermore, extensive and regular removal of possible mosquito breeding sites from the environment is necessary, especially the temporary containers.

## 5. Conclusion

Study results revealed 22 microbiota species belonging to nine phyla and a total of 26 microbiota species belonging to ten phyla from different mosquito breeding habitats of *Ae. aegypti* and *Ae. albopictus*, respectively. Larvae are not selecting all microbiota species as food items in which the microenvironment offers. Twelve microbiota species were recorded from larval gut of *Aedes aegypti* and *Ae. albopictus* up to a genus level, eight from each species. The distribution of gut microbiota species in both *Ae. aegypti* and *Ae albopictus* differed significantly at a 95% level of confidence. The findings of the current study would also be useful to identify the breeding habitats of dengue vectors and facilitate the implementation of appropriate vector control interventions. Identification of food items/associated microbiota led to focus on larval food limitation. Introduction of food competitors or indigestible food items for larvae could be a potential additional tool for integrated vector control approaches within the country. Identification of the food competing other biota, which prefer the same diet organisms as in mosquito larvae, is recommended for future studies.

## Figures and Tables

**Table 1:** Occurrence frequencies of microbiota species in different types of breeding habitats of *Ae. aegypti* and *Ae. albopictus.*

Species	Percentage occurrence in habitat type																
	Plastic containers		Tires		Gutters	Coconut shells		Ponds		Polythene waste	PVC tubes	Metal containers		Concrete slabs	Tree holes	Abandoned wells	Glassware
	*Ae. aegypti*	*Ae. albopictus*	*Ae. aegypti*	*Ae. albopictus*	*Ae. aegypti*	*Ae. aegypti*	*Aedes albopictus*	*Ae. aegypti*	*Ae. albopictus*	*Ae. aegypti*	*Ae. aegypti*	*Ae. aegypti*	*Ae. albopictus*	*Ae. albopictus*	*Ae. albopictus*	*Ae. albopictus*	*Ae. albopictus*
Phylum Ciliophora																	
*Paramecium caudatum*	—	—	45.5 (B)	—	—	—	—	—	—	—	—	39.2 (B).............	38. 9 (B).............	—	—	—	50.0 (B)
*Colpoda* sp.	—	—	—	—	—	—	—	—	—	18.4 (C)	—	—	—	—	—	—	—
*Paramecium bursaria*	—	—	—	45.5 (B)	—	—	—	—	—	—	—	—	—	—	—	—	—
Phylum Rotifera																	
*Cephalodella forficula*	—	—	—	—	—	—	—	—	—	—	—	—	—	—	—	7.7 (C)	—
*Monostyla hamata*	—	—	—	—	—	—	—	—	—	—	—	—	—	—	—	23.1 (C)	—
*Lecane luna*	12.5 (C)	—	—	—	—	23.5 (C)	62.5 (A)	—	4.1 (C)	—	—	—	—	—	—	—	—
*Philodina roseola*	—	—	—	—	—	11.8 (C)	—	—	—	—	—	—	—	—	—	—	—
*Brachionus urceus*	—	—	—	—	—	—	—	—	—	—	—	—	—	2.3 (C)	—	—	—
*Monostyla bulla*	—	—	—	—	—	29.3 (B)	—	—	—	—	10.6 (C)	—	9.7 (C)	—	—	—	29.2 (B)
*Notholca acuminata*	—	—	—	—	—	—	—	—	—	13.2 (C)	—	—	—	—	20.0 (C)	—	—
*Philodina citrina*	50.0 (B)	—	—	—	100 (A)	—	—	—	8.3 (C)	—	12.7 (C)	15.7 (C)	—	—	—	—	—
*Diurella stylata*	—	—	—	—	—	—	—	5.8 (C)	—	—	—	—	—	—	10.0 (C)	—	—
*Brachionus* sp.	—	—	—	—	—	—	—	—	—	—	4.3 (C)	—	—	—	—	—	—
*Euchlanis dilatata*	—	—	14.6 (C)	14.6 (C)	—	—	—	2.9 (C)	3.3 (C)	—	—	—	—	—	—	3.9 (C)	—
Phylum Cyanobacteria/Cyanophyta																	
*Spirulina major*	—	23.2(C)	—	—	—	11.8 (C)	37.5 (B)	—	—	—	19.2 (C)	—	—	—	—	—	—
*Anabaena affinis*	—	—	—	—	—	—	—	2.9 (C)	—	—	—	—	—	—	—	—	—
Phylum Amoebozoa																	
*Arcella arenaria*	—	—	21.8 (C)	21.8 (C)	—	11.8 (C)	—	—	—	42.1 (B)	—	19.6 (C)	—	—	—	—	—
Phylum Sarcodina																	
*Acanthocystis aculeata*	—	—	—	—	—	—	—	—	—	—	42.6 (B)	—	—	7.5 (C)	—	—	—
Phylum Euglenozoa																	
*Euglena pisiformis*	—	—	—	14.5 (C)	—	—	—	—	—	—	—	—	—	—	—	—	—
*Euglena acus*	—	—	14.6 (C)	—	—	—	—	—	—	—	—	—	—	—	—	19.2 (C)	—
*Phacus pleuronectes*	—	—	—	—	—	—	—	—	—	—	—	—	—	90.2 (A)	—	—	—
*Phacus caudatus*	—	—	—	—	—	—	—	36.2 (B)	—	26.3 (B)	—	—	13.9 (C)	—	—	—	20.8 (C)
Phylum Ochrophyta																	
*Pinnularia* sp.	—	—	3.5 (C)	3.6 (C)	—	—	—	—	—	—	—	—	2.8 (C)	—	60.0 (A)	—	—
Phylum Chlorophyta																	
*Crucigenia rectangularis*	—	—	—	—	—	—	—	—	12.4 (C)	—	—	—	—	—	—	—	—
*Gloeocystis gigas*	—	—	—	—	—	—	—	17.4 (C)	—	—	—	—	—	—	—	—	—
*Pediastrum biradiatum*	—	—	—	—	—	—	—	—	41.3 (B)	—	—	—	—	—	—	—	—
*Scenedesmus quadricauda*	—	—	—	—	—	—	—	2.9 (C)	—	—	—	—	—	—	—	—	—
*Scenedesmus armatus*	—	—	—	—	—	—	—	—	9.9 (C)	—	—	—	—	—	—	—	—
*Volvox aureus*	—	72.1 (A)	—	—	—	—	—	—	—	—	—	9.8 (C)	34.7 (B)	—	—	—	—
*Chlorella vulgaris*	—	—	—	—	—	—	—	—	16.5 (C)	—	—	—	—	—	—	38.4 (B)	—
*Scenedesmus bijuga*	—	—	—	—	—	—	—	—	4.2 (C)	—	—	—	—	—	10.0 (C)	—	—
Phylum Bacillariophyta																	
*Siurella* sp.	—	—	—	—	—	—	—	2.9 (C)	—	—	10.6 (C)	—	—	—	—	—	—
*Gomphonema angustatum*	37.5 (B)	—	—	—	—	11.8 (C)	—	29.0 (B)	—	—	—	15.7 (C)	—	—	—	—	—
*Synedra* sp.	—	—	—	—	—	—	—	—	—	—	—	—	—	—	—	7.7 (C)	—
Phylum Arthropoda																	
*Alona* sp.	—	4.7 (C)	—	—	—	—	—	—	—	—	—	—	—	—	—	—	—

A: constant species; B: common species; and C: accidental/ rare species of the samples are included in parenthesis.

**Table 2:** Evenness, Shannon diversity, alpha (*α*) medium, beta (*β*), and gamma (*ϒ*) diversities of type of habitats.

Breeding habitat	Mosquito sp.	No. of habitat positive for larvae	Alpha medium	Beta	Gamma	Shannon-Weiner diversity	Evenness
Plastic containers (*n* = 5)	*Ae. aegypti*	3	2	0.5	3	3.8	0.2
	*Ae. albopictus*	2	3	0	3	4.9	0.2
Tires (*n* = 4)	*Ae. aegypti*	1	5	0	5	9.5	0.5
	*Ae. albopictus*	3	3	0.7	5	9.5	0.5
Gutters (*n* = 3)	*Ae. aegypti*	3	1	0	1	0	0.00
Coconut shells (*n* = 3)	*Ae. aegypti*	2	3	0.5	6	11.2	0.5
	*Ae. albopictus*	1	2	0	2	1.5	0.1
Ponds (*n* = 3)	*Ae. aegypti*	1	8	0	8	21.0	1.0
	*Ae. albopictus*	2	4	0.5	8	19.4	0.9
Polythene waste (*n* = 3)	*Ae. aegypti*	3	3	0.3	4	5.9	0.3
PVC tubes (*n* = 3)	*Ae. aegypti*	3	6	0	6	12.2	0.6
Metal containers (*n* = 4)	*Ae. aegypti*	2	3	0.7	5	8.6	0.4
	*Ae. albopictus*	2	3	0.2	5	9.9	0.5
Concrete slabs (*n* = 3)	*Ae. albopictus*	3	2	0.5	3	6.5	0.3
Tree holes (*n* = 3)	*Ae. albopictus*	3	4	0	4	6.7	0.3
Abandoned wells (*n* = 3)	*Ae. albopictus*	3	6	0	6	9.9	0.5
Glassware (*n* = 3)	*Ae. albopictus*	3	3	0	3	3.5	0.2

**Table 3:** Occurrence of identified microbiota up to genus/species level, from the gut of *Ae. aegypti* and *Ae. albopictus* mosquito larvae.

Microbiota species	Percentage occurrence in mosquito species	
	*Ae. Aegypti*	*Ae. Albopictus*
Phylum Bacillariophyta		
*Cymbella affinis*	25.42	—
*Gomphonema angustatum*	13.56	—
*Synedra* sp.	—	3.13
Phylum Charophyta		
*Closterium* sp.	11.86	14.06
*Cosmarium quadrum*	—	7.81
Phylum Chlorophyta		
*Chlorella vulgaris*	—	28.13
*Volvox aureus*	—	12.50
Phylum Cyanobacteria/Cyanophyta		
*Oscillatoria* sp.	6.78	—
*Anabaena affinis*	11.86	15.62
*Spirulina major*	3.39	—
Phylum Euglenozoa		
*Phacus* sp.	6.78	3.13
Phylum Ochrophyta		
*Pinnularia* sp.	20.35	15.62

## Data Availability

The datasets supporting the conclusions of this article are included in the article.

## References

[1] Chaves L. F., Koenraadt C. J. M. (2010). Climate change and highland malaria: fresh air for a hot debate. *The Quarterly Review of Biology*.

[2] Simsek F. (2004). Seasonal larval and adult population dynamics and breeding habitat diversity of *Culex theileri* Theobald 1903 (Diptera: Culicidae) in the Golbasi district, Ankara. *Turkish Journal of Zoology*.

[3] Aigbodion F. I., Anyiwe M. A. (2005). Mosquitoes and environments: some economic costs of malaria in Nijeria. *Nijerian Journal of Entomology*.

[4] Overgaard H. J., Tsuda Y., Suwonkerd W., Takagi M. (2002). Characteristics of *Anopheles minimus* (Diptera: Culicidae) larval habitats in northern Thailand. *Environmental Entomology*.

[5] World health Organization, (WHO) Dengue fever – Sri Lanka, Disease Outbreak News, 19 July 2017. https://www.who.int/csr/don/19-july-2017-dengue-sri-lanka/en/.

[6] Chan K. L., Ho B. C., Chan Y. C. (1971). *Aedes aegypti* (L) and *Aedes albopictus* (Skuse) in Singapore city. *Bulletin of the World Health Organization*.

[7] Hawley W. A., Reiter P., Copeland R. S., Pumpuni C. B., Craig G. B. (1987). *Aedes albopictus* in North America: probable introduction in used tires from northern Asia. *Science*.

[8] Blaustein L., Chase J. M. (2007). Interactions between mosquito larvae and species that share the same trophic level. *Annual Review of Entomology*.

[9] Alfonzo D., Grillet M. E., Liria J., Navarro L. C., Weaver S. C., Barrera R. (2005). Ecological characterization of the aquatic habitats of mosquitoes (Diptera: Culicidae) in enzootic foci of Venezuelan equine encephalitis virus in western Venezuela. *Journal of Medical Entomology*.

[10] Beketov M. A., Liess M. (2007). Predation risk perception and food scarcity induce alterations of life-cycle traits of the mosquito *Culex pipiens*. *Ecological Entomology*.

[11] Knoechel R., Holtby L. B. (1986). Cladoceran filtering rate: body length relationship for bacterial and large algal particles. *Limnology and Oceanography*.

[12] Dole-Olivier M. J., Galassi D. M. P., Marmonier P., Creuzé Des Châtelliers M. (2000). The biology and ecology of lotic microcrustaceans. *Freshwater Biology*.

[13] Mokany A., Shine R. (2003). Biological warfare in the garden pond: tadpoles suppress the growth of mosquito larvae. *Ecology Entomology*.

[14] Saab S. A., Dohna H. z., Nilsson L. K. J. (2020). The environment and species affect gut bacteria composition in laboratory co-cultured *Anopheles gambiae* and *Aedes albopictus* mosquitoes. *Scientific Reports*.

[15] Nilsson L. K. J., de Oliveira M. R., Marinotti O. (2019). Characterization of bacterial communities in breeding waters of *Anopheles darlingi* in Manaus in the Amazon Basin malaria-endemic area. *Microbial Ecology*.

[16] Merritt R. W., Dadd R. H., Walker E. D. (1992). Feeding behavior, natural food, and nutritional relationships of larval mosquitoes. *Annual Review of Entomology*.

[17] Washburn J. O. (1995). Regulatory factors affecting larval mosquito populations in container and pool habitats: implications for biological control. *Journal of the American Mosquito Control Association*.

[18] Amarasinghe L. D., Rathnayake A. R. L. K. (2014). Prevalence of microfauna associated with different mosquito breeding habitats in a selected area of Sri Lanka. *International Journal of Current Microbiology and Applied Sciences*.

[19] Amarasinghe L. D., Ranasinghe H. A. K. (2019). Diversity and species composition of microbiota associated with mosquito breeding habitats: a study from Kurunegala district in Sri Lanka. *BioMed Research International*.

[20] Ranasinghe H. A. K., Amarasinghe L. D. (2020). Naturally occurring microbiota associated with mosquito breeding habitats and potential parasitic species against mosquito larvae: a study from Gampaha District, Sri Lanka. *BioMed Research International*.

[21] Epidemiology Unit, Ministry of Health (2019). *Sri Lanka: Disease surveillance*.

[22] Amerasinghe F. P. (1995). Illustrated keys to the genera of mosquitoes (Diptera: Culicidae) in Sri Lanka. *Journal of National Science Council of Sri Lanka*.

[23] Chelliah R. V. (1984). Keys and illustrations to the genera of mosquitoes of Sri Lanka (Diptera: Culicidae). *Contributions to American Entomological Institute*.

[24] Rattanarithikul R., Harrison B. A., Panthusiri P., Coleman R. E. (2005). Illustrated keys to the mosquitoes of Thailand 1, background; geographic distribution; list of genera, subgenera, and species; and a key to the genera. *The Southeast Asian Journal of Tropical Medicine and Public Health*.

[25] Rueda L. M. (2004). Pictorial keys for the identification of mosquitoes (Diptera: Culicidae) associated with dengue virus transmission. *Zootaxa*.

[26] Fernando C. H., Weerawardhena S. R. (2002). *A guide to the freshwater fauna of Ceylon (Sri Lanka)*.

[27] Abeywickrama B. A., Abeywickrama L. (1979). The genera of the freshwater algae of Sri Lanka. *Part 1,-UNESCO Man and the Biosphere National Committee for Sri Lanka*.

[28] Corliss J. O. (1979). *The Ciliated Protozoa: Characterization, Classification, and Guide to the liteBPrature*.

[29] Lobo E., Leighton G. (1986). Estructuras comunitarias de las fitocenosis planctonicas de los sistemas de desembocaduras de rios y esteros de la zona central de Chile. *Revista de Biologia Marina y Oceanografia*.

[30] Harrison S., Ross S. J., Lawton J. H. (1992). Beta diversity on geographic gradients in Britain. *Journal of Animal Ecology*.

[31] Nabout J. C., de Nogueira I. S., de Oliveira L. G., Morais R. R. (2007). Phytoplankton diversity (alpha, beta, and gamma) from the Araguaia River tropical floodplain lakes (central Brazil). *Hydrobiologia*.

[32] Shannon C. E., Weaver W. (1963). *The Mathematical Theory of Communication*.

[33] Pielou E. C. (1975). *Ecological Diversity*.

[34] Marten G. G. (2007). Larvicidal Algae. Biorational Control of Mosquitoes. *Journal of the American Mosquito Control Association*.

[35] Walker E. D., Olds E. J., Merritt R. W. (1988). Gut content analysis of mosquito larvae (Diptera: Culicidae) using DAPI stain and Epifluorescence microscopy. *Journal of Medical Entomology*.

[36] Dieng H., Mwandawiro C., Boots M. (2002). Leaf litter decay process and the growth performance of *Aedes albopictus* larvae (Diptera: Culicidae). *Journal of Vector Ecology*.

[37] Marten G. G. (1986). Mosquito control by plankton management: the potential of indigestible green algae. *The Journal of Tropical Medicine and Hygiene*.

[38] Marten G. G. (1984). Impact of the copepod *Mesocyclops leuckarti pilosa* and the green alga *Kirchneriella irregularis* upon larval *Aedes albopictus* (Diptera: Culicidae). *Bull Soc. Vector Ecol.*.

[39] Duguma D., Kaufman M. G., Simas Domingos A. B. (2017). Aquatic microfauna alter larval food resources and affect development and biomass of West Nile and Saint Louis encephalitis vector *Culex nigripalpus* (Diptera: Culicidae). *Ecology and Evolution*.

[40] Thiery I., Nicolas L., Rippka R., Tandeau de Marsac N. (1991). Selection of cyanobacteria isolated from mosquito breeding sites as a potential food source for mosquito larvae. *Applied and Environmental Microbiology*.

[41] Garcia-Sánchez D. C., Pinilla G. A., Quintero J. (2017). Ecological characterization of *Aedes aegypti* larval habitats (Diptera: Culicidae) in artificial water containers in Girardot, Colombia. *Journal of Vector Ecology*.

[42] Meyabeme Elono A. L., Liess M., Duquesne S. (2010). Influence of competing and predatory invertebrate taxa on larval populations of mosquitoes in temporary ponds of wetland areas in Germany. *Journal of Vector Ecology*.

